# Prevalence of Falls and Fractures in Alzheimer’s Patients Compared to General Population

**DOI:** 10.7759/cureus.12923

**Published:** 2021-01-26

**Authors:** Kapeel Dev, Alizay Javed, Priya Bai, Manish Murlidhar, Sidra Memon, Owais Alam, Zoha Batool

**Affiliations:** 1 Internal Medicine, Ghulam Muhammad Mahar Medical College, Sukkur, PAK; 2 Internal Medicine, Jinnah Postgraduate Medical Centre, Karachi, PAK; 3 Internal Medicine, People’s University of Medical and Health Sciences for Women, Nawabshah, PAK; 4 Internal Medicine, Chandka Medical College, Larkana, PAK; 5 Internal Medicine, Jinnah Sindh Medical University, Karachi, PAK

**Keywords:** falls, fractures, alzheimer’s

## Abstract

Introduction

Alzheimer’s disease (AD), found in the aging elderly population, is a progressive neurodegenerative disorder that leads to worsening memory loss and cognitive impairment. Falls and fractures are common in the overall elderly population. Hence, the purpose of this study is to determine the prevalence of falls and fractures in Alzheimer’s patients compared to the general population.

Methodology

This longitudinal study was conducted at the neurology outpatient department (OPD) in a tertiary healthcare setup in Pakistan from November 2019 till April 2020. Previously confirmed diagnosed Alzheimer’s patients from neurology OPD were included in one group. Equal number of gender and age-matched healthy participants were included in the reference group. Participants were followed for 12 months to determine the incidence of falls and non-vertebral fractures.

Results

The incidence of fall was significant in the Alzheimer group compared to the reference group (22.8% vs. 10.9%; relative risk (RR): 2.08; P-value: 0.01). Fractures were also significantly more common in the Alzheimer group compared to the reference group (12.8% vs. 5.1%; RR: 2.51; P-value: 0.03).

Conclusion

This study demonstrated a higher incidence of falls and fractures in Alzheimer’s patients compared to healthy non-Alzheimer individuals. Management of AD should include measures to reduce falls and fractures in addition to standard therapy.

## Introduction

Alzheimer’s disease (AD) in a neurodegenerative disorder of the elderly population manifested with marked loss of memory and cognitive abilities and noticeable changes in personality [1]. Globally, AD has an estimated prevalence of 10-30% in people aged above 65 years and an incidence of 1-3% with a long preclinical course. The disease is attributed to the deposition of amyloid-beta peptides in the brain among patients with sporadic and inherited genetic factors [2]. AD is a lethal variant of dementia, being the sixth leading cause of death in the United States [3].

Falls have a significant contribution to morbidity and mortality among the elderly, with an annual incidence of 30-40% in the population aged above 65 years. Falls lead to 87% of all fractures in the elderly [4]. Various studies suggest that dementia and neurocognitive impairment contribute to the prevalence of falls and fractures among elderly patients. A study published in 2018 showed that 31.1% of patients with dementia had a fall during a mean follow-up of 2.5 years and 17.7% suffered a fracture. Another study indicated that the prevalence of dementia was significantly higher among patients with hip fracture [5,6].

Falls and fractures pose a massive burden on the healthcare system, especially among the elderly population with preexisting diseases, which necessitates the need to assess and minimize the risk factors. In this study, we aim to compare the prevalence of falls and fractures among patients suffering from AD with the general population. This will help the physicians in understanding the magnanimity of the risk and devise appropriate measures to reduce the burden.

## Materials and methods

This longitudinal study was conducted at the internal medicine department from November 2019 to April 2020. A total of 150 Alzheimer’s patients with previously confirmed diagnoses, with the help of clinical symptoms and magnetic resonance imaging (MRI) scan, were included from the neurology outpatient department after obtaining informed consent from the patient and/or attendant. Patients were on cholinesterase inhibitor drugs. Gender and age-matched 150 healthy participants were included in the study as the reference group. Age, gender, smoking status, body mass index, and physical activity were noted in a self-structured questionnaire. Physical activity was defined as one to two days of exercise in one week. Instituitional Review Board approval was also sought before starting the study.

Participants were followed for 12 months to determine the incidence of falls and non-vertebral fracture. Participants and/or their attendants were called on phone every month to inquire about any fall or fracture in the last 30 days. A total of six patients were lost to follow-up in the Alzheimer’s group and 10 participants were lost to follow-up in the reference group. A total of four patients died in the Alzheimer’s group and three patients died in the reference group. A total of 140 participants completed the study in the Alzheimer’s group, while 137 participants completed the study in the reference group. Only participants who completed the study were included in the final analysis.

Statistical analysis was done using the Statistical Package for the Social Sciences version 23.0 (IBM Corporation, Armonk, New York, United States). Continuous variables were analyzed via descriptive statistics and were presented as means and standard deviation (SD) while categorical variables were presented as percentages and frequencies. T-test and Chi-square test were applied as appropriate. Relative risk (RR) was used to compare patients in the AD group with the reference group. P-value of less than 0.05 meant that there was significant difference between the two groups and null hypothesis was void.

## Results

Characteristics were comparable between both groups, except participants were more physically active in the reference group (Table 1).

**Table 1:** Comparison of characteristics between the Alzheimer’s and reference groups. SD: standard deviation; NS: non-significant; BMI: body mass index

Characteristics	Alzheimer’s Group (n = 140)	Reference Group (n = 137)	P-Value
Age in years (Mean ± SD)	65 ± 10	63 ± 11	NS
Male (%)	52	48.17	NS
BMI greater than 25 kg/m^2^ (%)	44.2	43.7	NS
Current smokers (%)	14.2	25.7	NS
Physically active (%)	14.2	16.1	0.005

The incidence of fall was significantly more in the Alzheimer’s group compared to the reference group (22.8% vs. 10.9%; RR: 2.08; P-value: 0.01). Fractures were also significantly more common in the Alzheimer’s group compared to the reference group (12.8% vs. 5.10%; RR: 2.51; P-value: 0.03) (Table 2).

**Table 2:** Comparison of outcomes between the Alzheimer’s and reference groups. NS: non-significant; CI: confidence interval

Outcome	Alzheimer’s Group (n = 140)	Reference Group (n = 137)	Relative Risk (95%, CI)	P-Value
Minimum of one fall	32 (22.8%)	15 (10.9%)	2.08 (1.18-3.67)	0.01
Multiple falls	8 (5.7%)	2 (1.45%)	3.91 (0.84-18.100)	NS
Non-vertebral fracture	18 (12.8%)	7 (5.10%)	2.51 (1.08-5.83)	0.03

## Discussion

Various studies have identified older age, history of previous falls, the necessity of walking aid, presence of visual impairment, psychotropic drugs, balance disorders, and functional impairment as major risk factors of falls in the elderly population [4,7-11]. Some of these risk factors have also been identified in patients with dementia, especially those suffering from AD [12-14] Motor impairment signs such as gait disturbances, extrapyramidal motor impairment, rigidity, and postural instability, as well as behavioral disturbances such as attentional deficits, wandering, and aggressiveness have been strongly associated with falls and fractures in people with dementia or AD [14-16]. Dyer et al. analyzed the data from an 18-month randomized-controlled trial of nilvadipine in mild-to-moderate AD (NILVAD) and concluded that there was a significant association between poorer cognition and slow gait speed, which was associated with a significantly increased risk of falls in patients with mild-to-moderate AD [17]. Horikawa et al. predicted that high grades of periventricular white matter lesions and the use of neuroleptic drugs were significantly associated with a high risk of falls in patients with AD [13].

The prevalence of falls and non-vertebral fractures in Alzheimer’s patients was compared with the general population in our study. The present study included 140 participants in the Alzheimer’s group and 137 participants in the reference group. Incidence of a minimum of one fall (22.8% vs. 10.9%; P-value: 0.01) was significantly higher in patients with AD compared to the healthy individuals in the reference group. However, the incidence of multiple falls was not significant (5.7% vs 1.45%). This may be due to lack of movement after the first fracture.

Kato-Narita et al. reported the incidence of at least one fall over a period of 12 months to be 56% in patients with mild AD and 55% in patients with moderate AD. Environmental hazard risks were identified as the major cause of falls in both mild and moderate AD while falls due to dizziness and instability were more common in patients with mild AD [14]. Buchner et al. in their study to predict fall and fractures rate in patients with Alzheimer’s type-dementia reported that 50% of the patients had a fall during a three-year follow-up. These falls were reported more in patients who experienced toxic reactions to drugs on entry into the study [18].

The present study also concluded that the incidence of non-vertebral fractures (12.8% vs. 5.83%; P-value: 0.03) was also significantly higher in patients with AD compared to the healthy individuals in the reference group. Buchner et al. reported fracture rates, including those involving the hip, to be three times higher in AD patients than the age- and sex-adjusted fracture rate in the general population, and these fractures were more commonly found in AD patients who wandered [18]. In a meta-analysis including nine studies, Zhao et al. concluded that AD patients have a low bone mineral density, a predictor of fracture, and are at a higher risk of developing hip fracture [19].

To the best of our knowledge, this is the first study in Pakistan that compares the prevalence of falls and fractures in Alzheimer’s patients with the general population. The sample was included from one institute, which might have made the sample less diversified. Another limitation of the study was that due to lack of data, we were not able to correlate the severity of AD and falls.

## Conclusions

Incidence of fall and non-vertebral fractures were significantly more common in patients with AD compared to the general population. Various risk factors have been identified in literature that are responsible for increase in fall and fractures in AD such as older age, use of psychotropic drugs, motor impairment, and behavioral symptoms. It is important that caretakers of AD patients should be made aware of the increase in the risk of fall and fracture and necessary measures should be taken to prevent fractures and falls.
